# *MLH1* Promoter Methylation Frequency in Colorectal Cancer Patients and Related Clinicopathological and Molecular Features

**DOI:** 10.1371/journal.pone.0059064

**Published:** 2013-03-29

**Authors:** Xia Li, Xiaoping Yao, Yibaina Wang, Fulan Hu, Fan Wang, Liying Jiang, Yupeng Liu, Da Wang, Guizhi Sun, Yashuang Zhao

**Affiliations:** 1 Department of Epidemiology, Public Health College, Harbin Medical University, Harbin, Heilongjiang Province, P.R. China; 2 Department of Science and Technology Administration, Harbin Medical University, Harbin, Heilongjiang Province, P.R. China; 3 Department of Surgery, Cancer Hospital of Harbin Medical University, Harbin, Heilongjiang Province, P.R. China; The Chinese University of Hong Kong, Hong Kong

## Abstract

**Purpose:**

To describe the frequency of *MLH1* promoter methylation in colorectal cancer (CRC); to explore the associations between *MLH1* promoter methylation and clinicopathological and molecular factors using a systematic review and meta-analysis.

**Methods:**

A literature search of the PubMed and Embase databases was conducted to identify relevant articles published up to September 7, 2012 that described the frequency of *MLH1* promoter methylation or its associations with clinicopathological and molecular factors in CRC. The pooled frequency, odds ratio (OR) and 95% confidence intervals (95% CI) were calculated.

**Results:**

The pooled frequency of *MLH1* promoter methylation in unselected CRC was 20.3% (95% CI: 16.8–24.1%). They were 18.7% (95% CI: 14.7–23.6%) and 16.4% (95% CI: 11.9–22.0%) in sporadic and Lynch syndrome (LS) CRC, respectively. Significant associations were observed between *MLH1* promoter methylation and gender (pooled OR = 1.641, 95% CI: 1.215–2.215; *P* = 0.001), tumor location (pooled OR = 3.804, 95% CI: 2.715–5.329; *P*<0.001), tumor differentiation (pooled OR = 2.131, 95% CI: 1.464–3.102; *P*<0.001), MSI (OR: 27.096, 95% CI: 13.717–53.526; *P*<0.001). Significant associations were also observed between *MLH1* promoter methylation and *MLH1* protein expression, *BRAF* mutation (OR = 14.919 (95% CI: 6.427–34.631; *P*<0.001) and 9.419 (95% CI: 2.613–33.953; *P* = 0.001), respectively).

**Conclusion:**

The frequency of *MLH1* promoter methylation in unselected CRC was 20.3%. They were 18.7% in sporadic CRC and 16.4% in LS CRC, respectively. *MLH1* promoter methylation may be significantly associated with gender, tumor location, tumor differentiation, MSI, *MLH1* protein expression, and *BRAF* mutation.

## Introduction

Colorectal cancer (CRC) is one of the most common malignancies, representing the third most common cancer in men and the second in women worldwide [1]. One of the genetic pathways in the development of CRC is the microsatellite instability (MSI) [2].

Microsatellites are repeated DNA sequences which occur approximately every 50–100 Kb base pairs throughout the human genome [3], [4]. Multiple studies have indicated that about 90% of the Lynch Syndrome (LS) [5], [6] and 10% to 15% of sporadic CRC can be detected of MSI [3], [4]. MSI in LS and sporadic CRC occurs through two different mechanisms. In LS, MSI is mainly caused by germline mutation of mismatch repair genes [7]. MSI in sporadic CRC is commonly due to methylation induced silencing of the *MLH1* gene [8].

DNA methylation refers to the presence of a methyl group on a cytosine residue [9]. DNA methylation of tumor suppressor genes leading to transcriptional inactivation has been identified as an important mechanism in human carcinogenesis [10], [11]. *MLH1* gene, as a number of suppressor genes, is prone to be silenced by promoter methylation in CRC [8], [12], [13].

Since the first report of *MLH1* promoter methylation in sporadic colon tumors [8], the prevalence of *MLH1* promoter methylation have been widely studied not only in sporadic but also in LS CRC. However, the results are inconsistent. The frequency of *MLH1* promoter methylation in sporadic CRC varied from 0.0% [14] to 66.9% [15]. It varied from 0.0% [16] to 21.4% [17] in LS CRC.

*BRAF* and *KRAS* are important members of RAS/RAF/MAPK signaling pathway, which regulates cell growth, proliferation, differentiation, and apoptosis in malignant and nonmalignant cells [18]. *BRAF* mutation has been shown to be associated with *MLH1* promoter methylation [19], [20]. Whereas, *MLH1* promoter methylation was few detected in *KRAS* mutant CRC [21]. The associations between *MLH1* promoter methylation and *BRAF* and *KRAS* mutation in CRC have been widely studied with inconsistent results [15], [21], [22], [23]. The associations between *MLH1* promoter methylation and other clinicopathological and molecular characteristics of CRC such as tumor location, tumor staging, tumor differentiation, family history, MSI, and *MLH1* protein expression were also widely studied. However, the results are inconsistent. Therefore, we conducted a systematic review and meta-analysis to accurately estimate the frequency of *MLH1* promoter methylation in LS and sporadic CRC, and the associations between *MLH1* promoter methylation and clinicopathological/molecular characteristics of CRC.

## Methods

### Search Strategy and Selection Criteria

We conducted a systematic literature search using PubMed and Embase from January 1, 1997 to September 7, 2012 to identify all the relevant English-language articles. The following keywords were used: “methylation” and “*MLH1*” and “promoter” and “colorectal cancer” and/or “carcinoma” or “tumor” or “neoplasm”. We also hand-searched the reference lists of the retrieved articles and reviews for additional articles.

The inclusion/exclusion criteria were as follows: (1) papers on *MLH1* promoter methylation in unselected CRC were included. In contrast, papers that selected subgroups were excluded (such as selected based on age, tumor staging and ulcerative colitis-associated CRC); (2) sporadic CRC and/or LS related CRC remained as specific selected groups, often stratified by MSI status and/or *MLH1* expression loss; (3) data regarding the DNA methylation of tumor tissue of CRC were included in the pooled analysis, whereas data regarding the DNA methylation of normal colonic mucosa [24], [25], [26], serum [27], [28], and peripheral blood leukocyte [29], [30], [31] of CRC were excluded; (4) studies that investigated multiple CRCs were excluded [32], [33], [34]; (5) case reports were excluded; (6) repetitive reports were unified by using the latest or the largest edition; (7) paper with insufficient or duplicated data were excluded.

### Data Extraction

Two authors (X. and X.P) independently conducted literature searches to identify all possible papers that met the inclusion criteria. Disagreements were settled by consensus or a third review (Y.B.N) for adjudication. The following information were extracted from every eligible study: authors, publication year, continent, country, patient source, sample size, methylation detecting method, positive frequency, gender, family history, tumor location (proximal and distal), tumor staging, and promoter regions.

### Classification of Family History

Patients had no family history of cancer regardless of the onset age were categorized as sporadic CRC. LS was diagnosed if a patient with family history met either Amsterdam criteria (I or II) [35], [36] or Bethesda criteria (original or revised) [37], [38] or confirmed with germline mutation in a DNA mismatch repair gene [39], [40]. The unselected CRC tumors were defined as patients from nature population or hospital-based. The unselected CRC tumors included sporadic and LS CRC, which were defined as total CRC.

### Tumor Staging and Differentiation

Tumor staging was categorized as I, II, III and IV stages based on the TNM classification (The Union for International Cancer Control [UICC]) [41]. Stage I: Cancer has begun to spread, but is still in the inner lining. Stage II: Cancer has spread to other organs near the colon or rectum. It has not reached lymph nodes. Stage III: Cancer has spread to lymph nodes, but has not been carried to distant parts of the body. Stage IV: Cancer has been carried through the lymph system to distant parts of the body. Differentiation was graded on a scale of poor, moderate or well differentiation.

### Promoter Regions

Promoter regions tested were noted as the A, B, C and D regions proposed by Deng et al. [42], where primer sequences were given. Promoter regions were checked against the sequence −1000 to −1, relative to the start codon of *MLH1*.

### Molecular Classification

MSI is typically assessed by analyzing five microsatellite markers (BAT25, BAT26, D2S123, D5S346, and D17S250) suggested by the National Cancer Institute [43]. One study expanded this panel to ten markers, which made the diagnosis of MSI CRC easier [38]. In this meta-analysis, three categories of MSI status were defined according to the following criteria: two or more loci out of five loci with instability (or ≥30–40% of loci if a larger panel of markers was used) was defined as MSI-H; one locus with instability (or <30–40% of loci in larger panels) was defined as lower-level microsatellite instability (MSI-L); and no loci with instability (or no apparent instability in larger panels) was defined as microsatellite stable (MSS). For papers without detailed information about MSI-H and MSI-L, only two levels of microsatellite instability status could be categorized: MSI-positive (MSI) and MSI-negative (MSS). *BRAF* and *KRAS* status were classified into mutant and wild type. *MLH1* protein expression status was defined as positive or negative.

### Statistical Analysis

The pooled frequency of *MLH1* promoter methylation and 95% confidence intervals (95% CI) were estimated. The frequency of *MLH1* promoter methylation was compared in different tumor characteristics. Heterogeneity among studies was evaluated with Cochran’s Q test [44] and the *I^2^* statistic [45], [46]. When heterogeneity was not an issue (*I^2^* values <50%), a fixed effect model was used to calculate parameters. If there was substantial heterogeneity (*I^2^* values ≥50%), a random-effects model was used to pool data and attempt to identify potential sources of heterogeneity based on subgroup analyses. The pooled OR was estimated for the association between *MLH1* promoter methylation and clinicopathological, molecular features. *P* values tailed less than 0.05 were considered statistically significant.

Publication bias was evaluated with funnel plot, Begg’s rank correlation [47], and Egger’s regression [48]. If publication bias existed, the trim and fill method was used to adjust the pooled frequency, pooled OR and 95% CI [49]. Data were calculated with Comprehensive Meta-Analysis V2.

## Results

**Figure 1:**
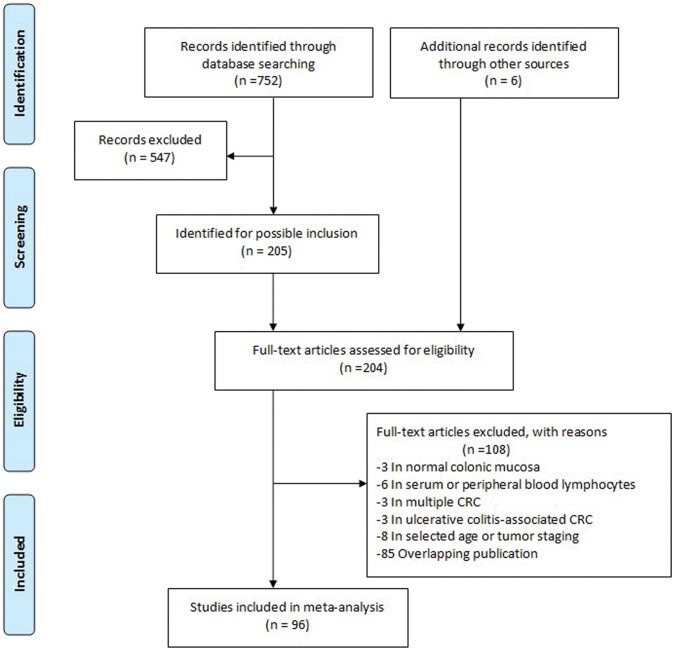
Flow diagram of study selection.

752 relevant articles were identified for initial review according to the inclusion and exclusion criteria. After screening, information for 10528 individuals from 96 studies was reviewed and included in the meta-analyses. Figure 1 showed the detailed selection process of articles.

### Frequency of *MLH1* Promoter Methylation in Total CRC Tumors

**Figure 2:**
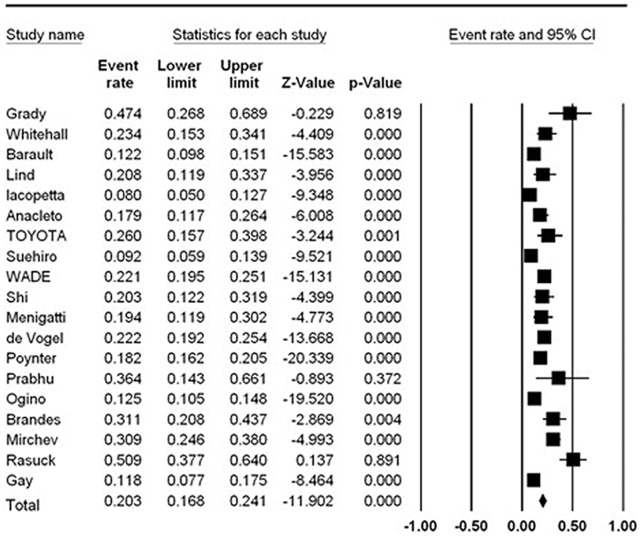
The pooled frequency of *MLH1* promoter methylation in CRC.

Overall, there were 19 studies with 5584 patients demonstrating *MLH1* promoter methylation in total CRC tumors. The total frequency of *MLH1* promoter methylation was 20.3% (95% CI: 16.8–24.1%) (Table 1, Figure 2). There was significant heterogeneity among the studies (*I^2^* = 87.896%).

**Table 1:** Pooled frequency of *MLH1* promoter methylation in colorectal cancer patients with different clinicopathological features.

Classification	No. ofstudies	No. ofdetectedcases	No. ofmethylationcases	Pooled frequencyand 95%CI (%)	*P*	Heterogeneity		Publication bias	
						*I^2^* (%)	*P*	Begg’stest *P*	Egger’stest *P*
CRC(total)	19	5584	1005	20.3(16.8–24.1)*	0.000	87.896	0.000	0.132	0.472
Subject source**									
Hospital-based	16	2335	449	20.5(15.6–26.5)*	0.000	86.938	0.000	0.589	0.761
Population-based	4	3249	556	16.4(12.1–22.0)*	0.000	93.166	0.000	0.497	0.244
Family history									
LS	8	243	31	16.4(11.9–22.0)	0.000	15.564	0.165	0.048	0.001
SCRC	29	3583	739	18.7(14.7–23.6)*	0.000	90.505	0.000	0.143	0.032
Gender**									
Female	6	555	124	20.8(15.6–27.2)	0.000	48.843	0.082	0.851	0.255
Male	6	699	102	11.8(6.9–16.5)*	0.000	75.322	0.001	0.348	0.136
Location**									
Proximal	6	474	139	29.6(20.4–40.8)*	0.001	76.757	0.001	0.573	0.795
Distal	6	698	71	6.5(3.0–13.4)*	0.000	74.442	0.002	0.348	0.029
UICC stage**									
I & II	4	160	34	22.4(15.6–31.0)	0.000	20.902	0.285	0.174	0.028
III & IV	4	123	24	25.5(9.3–53.5)*	0.083	82.686	0.001	1.000	0.807
Differentiation**									
Poor	6	182	56	31.0(24.6–38.1)	0.000	0.000	0.637	0.188	0.540
Moderate or Well	6	769	130	17.6(11.9–25.3)*	0.000	68.101	0.008	0.188	0.796

Abbreviations: CRC, colorectal cancer; LS, lynch syndrome; SCRC, sporadic colorectal cancer.

* Random effect estimate.

** It is only pooled data with total colorectal cancer.

### Family History

The frequency of *MLH1* promoter methylation was 18.7% (95% CI: 14.7–23.6%) in 3583 sporadic CRC of 29 studies and 16.4% (95% CI: 11.9–22.0%) in 243 LS reported in eight studies (Table 1). The frequency of *MLH1* promoter methylation in sporadic MSI-H and LS CRC MSI-H were 73.6% (95% CI: 67.3–79.0%) and 15.3% (95% CI: 8.8–25.4%), respectively (Table 2). For MSI-H CRC, significant association between *MLH1* promoter methylation and family history was observed (pooled OR = 20.828, 95% CI: 4.056–106.950; *P*<0.001; *I^2^* = 55.363%; Table 3), when pooled data on four studies [50], [51], [52], [53].

**Table 2:** Pooled frequency of *MLH1* promoter methylation in colorectal cancer patients with different molecular features.

Classification	No. ofstudies	No. ofdetectedcases	No. ofmethylationcases	Frequency and95%CI (%)	Heterogeneity		Publication bias	
					*I^2^* (%)	*P*	Begg’stest *P*	Egger’stest *P*
CRC(total) MSI Status								
MSI-H	12	968	566	62.6(54.0–70.4)*	78.288	0.000	0.411	0.049
MSI-L	4	344	24	12.2(3.0–38.2)*	90.733	0.000	1.000	0.845
MSI	16	1325	576	55.8(45.2–65.8)*	89.197	0.000	0.280	0.002
MSS	10	1791	155	5.2(2.2–11.6)*	88.938	0.000	0.655	0.068
LS- MSI-H	5	95	11	15.3(8.8–25.4)	0.000	0.517	0.050	0.160
SCRC MSI Status								
MSI-H	11	249	188	73.6(67.3–79.0)	42.882	0.000	0.119	0.228
MSI	7	193	125	67.3(47.1–82.7)*	79.255	0.000	0.293	0.253
MSS	4	264	51	17.5(10.0–29.0)	35.109	0.202	0.174	0.014
CRC(total) MLH1expression								
Positive	2	67	7	11.9(1.5–53.8)*	83.531	0.014	–	–
Negative	5	106	73	66.5(44.4–83.2)*	73.534	0.004	1.000	0.981
MSI-H- Loss of *MLH1* protein	5	247	201	80.8(75.3–85.3)	0.000	0.644	0.624	0.321
SCRC*-MLH1* expression								
Positive	6	308	25	9.8(3.4–25.2)*	76.529	0.001	0.851	0.366
Negative	10	156	108	69.8(45.5–86.5)*	78.786	0.000	0.089	0.083
MSI-H- Loss of *MLH1* protein	3	64	56	86.3(75.3–92.9)	0.000	0.618	0.602	0.782
LS- Loss of *MLH1* protein	5	169	65	37.8 (25.3–52.1)*	62.057	0.000	0.142	0.155
CRC(total)*BRAF* situation								
Mutant	3	138	70	53.2(27.7–77.2)*	67.744	0.045	0.602	0.572
Wild type	3	764	113	13.7(5.1–32.0)*	90.433	0.000	0.602	0.792
CRC(total)*KRAS* situation								
Mutant	3	353	41	14.0(10.2–19.0)	9.722	0.330	0.117	0.119
Wild type	3	570	135	21.8(13.2–33.8)*	77.209	0.012	0.602	0.699

Abbreviations: CRC, colorectal cancer; LS, lynch syndrome; MMR, mismatch repair; SCRC, sporadic colorectal cancer; MSI, Microsatellite instability; MSS, microsatellite stability; MSI-L, lower-level microsatellite instability; MSI-H, high-level microsatellite instability.

* Random effect estimate.

**Table 3:** Pooled associations between *MLH1* promoter methylation and clinicopathological and molecular features.

Classification	No. of studies	OR and 95%CI	*P*	Heterogeneity		Publication bias	
				*I^2^* (%)	*P*	Begg’s test *P*	Egger’s test *P*
SCRC- MSI-H vs. LS- MSI-H	4	20.828(4.056–106.950)*	0.000	55.363	0.081	0.497	0.225
Female vs. Male**	6	1.641(1.215–2.215)	0.001	33.819	0.183	0.851	0.438
Proximal vs. Distal**	6	3.804(2.715–5.329)	0.000	46.541	0.096	0.348	0.042
UICC stage (I & II vs. III & IV)**	4	1.044(0.441–2.471)	0.922	42.854	0.154	0.497	0.773
Poor vs. Moderate or Well**	6	2.131(1.464–3.102)	0.000	0.000	0.674	0.573	0.430
MSI vs. MSS**	10	27.096(13.717–53.526)*	0.000	59.001	0.000	0.531	0.438
MSI-H vs. Non- MSI-H**	4	17.061(3.850–75.610)*	0.000	69.023	0.021	0.497	0.336
MSI vs. MSS***	4	33.549(3.942–285.515)*	0.001	81.145	0.001	0.497	0.297
*MLH1* expression***							
Negative vs. Positive	6	14.919(6.427–34.631)	0.000	35.469	0.171	0.573	0.455
*BRAF* situation							
Mutant vs. Wild type**	3	9.419(2.613–33.953)*	0.001	67.030	0.048	0.602	0.115
CRC- MSI-H**							
Mutant vs. Wild type	3	37.615(10.011–141.311)	0.000	0.000	0.913	0.117	0.251
*KRAS* situation							
Mutant vs. Wild type**	3	0.476(0.322–0.703)	0.000	49.293	0.139	0.117	0.161
MSI- CRC**							
Mutant vs. Wild type	2	0.340(0.167–0.693)	0.003	0.000	0.674	–	–

Abbreviations: CRC, colorectal cancer; LS, lynch syndrome; SCRC, sporadic colorectal cancer; MSI, Microsatellite instability; MSS, microsatellite stability;

MSI-H, high-level microsatellite instability.

* Random effect estimate.

** It is only pooled data with total colorectal cancer.

*** It is only pooled data with sporadic colorectal cancer.

### Gender

Gender information was available for 6 of the 19 studies with a total of 555 female and 699 male patients [16], [21], [22], [54], [55], [56]. The *MLH1* promoter methylation in female and male were 20.8% (95% CI: 15.6–27.2%) and 11.8% (95% CI: 6.9–16.5%) in total CRC group (pooled OR = 1.641, 95% CI = 1.215–2.215; *P = *0.001; *I^2^* = 33.819%) (Table 1 and Table 3).

### Tumor Location

*MLH1* promoter methylation was observed in 29.6% (95% CI: 20.4–40.8%) of the 474 proximal tumors and 6.5% (95% CI: 3.0–13.4%) of the 698 distal tumors in six studies (Table 1). Significant association was observed between *MLH1* promoter methylation and tumor location (pooled OR = 3.804, 95% CI: 2.715–5.329; *P*<0.001; *I^2^* = 46.541%) (Table 3). These data were based on six studies covering a total of 1172 patients (Table 1).

### Tumor Staging

The pooled prevalence of *MLH1* promoter methylation and pooled OR for the association between *MLH1*promoter methylation and the UICC stage were estimated in four studies [54], [56], [57], [58] (Table 1 and Table 3). The pooled prevalence of *MLH1* promoter methylation in stages I & II and in stages III & IV were 22.4% (95% CI: 15.6–31.0%) and 25.5% (95% CI: 9.3–53.5%), respectively (Table 1). The association between *MLH1* promoter methylation and tumor staging was not significant (pooled OR = 1.044, 95% CI: 0.441–2.471; *P = *0.922; *I^2^* = 42.854%) (Table 3).

### Tumor Differentiation

Six studies [16], [21], [54], [55], [56], [57] addressed the frequency of *MLH1* promoter methylation in total CRC according to tumor differentiation. The frequency of *MLH1* promoter methylation was 31.0% (95% CI, 24.6–38.1%) in 182 poor-differentiated CRC and 17.6% (95% CI, 11.9–25.3%) in 769 moderate or well-differentiated CRC, respectively (Table 1). *MLH1* promoter methylation in poor-differentiated CRC was significantly higher than in moderate or well-differentiated CRC (pooled OR = 2.131, 95% CI, 1.464–3.102; *P*<0.001; *I^2^* = 0.000%) (Table 3).

### Microsatellite Instability

For total CRC, *MLH1* promoter methylation was detected in 62.6% (95% CI: 54.0–70.4%) of the 968 MSI-H CRC in 12 studies, 12.2% (95% CI: 3.0–38.2%) of the 344 MSI-L CRC in four studies, 55.8% (95% CI: 45.2–65.8%) of the 1325 MSI CRC in 16 studies, and 5.2% (95% CI: 2.2–11.6%) of the 1791 MSS CRC in 10 studies (*P*<0.001), respectively (Table 2). Significant differences were found between MSI vs. MSS, MSI-H vs. MSS, and MSI-H vs. MSI-L (*P*<0.001, *P*<0.001, and *P*<0.001, respectively). Whereas, no difference was observed between MSI-L and MSS (*P* = 0.380). For sporadic CRC, the pooled prevalence of *MLH1* promoter methylation was 73.6% (95% CI: 67.3–79.0%) in MSI-H CRC, 67.3% (95% CI: 47.1–82.7%) in MSI CRC, and 17.5% (95% CI: 10.0–29.0%) in MSS CRC (*P*<0.001; Table 2). In addition, for the 10 studies [16], [22], [53], [54], [57], [59], [60], [61], [62], [63] that provided both MSI and MSS status in total CRC, the pooled OR for the association between *MLH1* promoter methylation and MSI status (MSI vs. MSS) was 27.096 (95% CI: 13.717–53.526; *P*<0.001; *I^2^* = 59.001%; Table 3, Figure S1).

### *MLH1* Protein Expression

In tumors with a loss of *MLH1* protein expression, *MLH1* promoter methylation was detected in 66.5% (95% CI: 44.4–83.2%) of the 106 total CRC, 80.8% (95% CI: 75.3–85.3%) of the 247 MSI-H CRC, 69.8% (95% CI: 45.5–86.5%) of the 156 sporadic CRC, and 37.8% (95% CI: 25.3–52.1%) of the 169 LS tumors (*P*<0.001). Significant differences were observed when comparing LS tumors vs. total CRC, LS tumors vs. sporadic CRC, and LS tumors vs. MSI-H CRC tumors (*P* = 0.032, *P*<0.001, and *P* = 0.026, respectively). For tumors with *MLH1* protein expression, *MLH1* promoter methylation was detected in 11.9% (95% CI: 1.5–53.8%) of the 67 total CRC and in 9.8% (95% CI: 3.4–25.2%) of the 308 sporadic CRC (*P* = 0.862; Table 2). Six studies [64], [65], [66], [67], [68], [69] provided expression status as a loss of *MLH1* protein expression in 308 cases and *MLH1* protein expression in 75 cases of sporadic CRC. The pooled analysis showed significantly association between *MLH1* promoter methylation and *MLH1* protein expression (OR = 14.919, 95% CI: 6.427–34.631%; *P*<0.001; *I^2^* = 35.469%) (Table 3).

### *BRAF* Mutation

The pooled prevalence of *MLH1* promoter methylation in 138 *BRAF*-mutated and 764 *BRAF* wild type CRC was 53.2% (95% CI: 27.7–77.2%) and 13.7% (95% CI: 5.1–32.0%) in three studies (pooled OR = 9.419; 95% CI: 2.613–33.953; *P = *0.001; *I^2^* = 67.030%) (Table 2 and Table 3). For the three studies that provided both MSI-H and *BRAF* mutation status in CRC, the pooled OR for the association between *BRAF* mutation status and *MLH1* promoter methylation was 37.615 in MSI-H CRC (95% CI: 10.011–141.311; *P*<0.001; *I^2^* = 0.000%) (Table 3).

### *KRAS* Mutation

The pooled frequency of *MLH1* promoter methylation was 14.0% (95% CI: 10.2–19.0%) in 353* KRAS*-mutated and 21.8% (95% CI: 13.2–33.8%) in 570 wild-type CRC, in three studies [15], [21], [22] (pooled OR = 0.476; 95% CI: 0.322–0.703; *P*<0.001; *I^2^* = 49.293%) (Table 2 and Table 3). Moreover, a statistically significant association was observed between *MLH1* promoter methylation and *KRAS* mutation in MSI CRC (OR = 0.340; 95% CI: 0.167–0.693; *P = *0.003; *I^2^* = 0.000%) (Table 3).

### Publication Bias

For the frequency of *MLH1* promoter methylation in MSI CRC and MSI-H CRC, the funnel plot seemed asymmetry (Figure S2 A and B). Funnel plot for the association between *MLH1* promoter methylation and tumor location (proximal vs. distal) also seemed asymmetry (Figure S3). Begg’s rank correlation and Egger’s regression methods further supported the significant publication bias. With the trim and fill method, the adjusted frequency of *MLH1* promoter methylation decreased from 55.8% to 36.7% in MSI CRC and from 62.6% to 53.5% in MSI-H CRC. The pooled OR for the association between *MLH1* promoter methylation and tumor location decreased from 3.804 (95% CI: 2.715–5.329) to 3.172 (95% CI: 2.323–4.331).

## Discussion

Our meta-analysis suggested that the frequency of *MLH1* promoter methylation in total CRC was 20.3%. They were 18.7% in sporadic CRC and 16.4% in LS CRC, respectively; significant associations were observed between *MLH1* promoter methylation and gender, tumor location, tumor differentiation, MSI, *MLH1* protein expression, and *BRAF* mutation.

The pooled *MLH1* promoter methylation frequencies were 16.4% and 20.5% in 4 population-based studies and 16 hospital-based studies (One study [53] included 1061 population-based and 172 hospital-based CRC). In total CRC, the frequency of the *MLH1* promoter methylation between hospital-based and population-based studies was not significantly different (*P* = 0.279) (Table 1).

A, B, C and D regions in the *MLH1* promoter were commonly tested for methylation. However, only one study tested the *MLH1* promoter methylation in “A” region [69], three studies tested the *MLH1* promoter methylation in “C” region [56], [57], [70], other 15 studies did not provide the specific A, B, C or D regions in total CRC. The *MLH1* promoter methylation frequency in “A” region (66.9%) was significantly higher than in “C” region in CRC (26.4%; *P* = 0.001) (Table S1). It may be due to the variation of methylation status in different regions of the *MLH1* promoter.

The *MLH1* promoter methylation was detected in total of 12 studies with 968 MSI-H CRC with a frequency of 62.6%. After the adjustment by trim and fill method, the pooled frequency decreased to 53.5%. The pooled *MLH1* promoter methylation in 249 sporadic MSI-H CRC was 73.6%, significantly higher than in 95 LS MSI-H CRC (15.3%). The following may explain our results: in sporadic CRC, MSI-H was mainly caused by *MLH1* promoter methylation [13], [71]; whereas, in LS CRC, MSI-H was mainly caused by MMR inactivation because of germline mutation [72].

In sporadic CRC, our meta-analysis indicated that the *MLH1* promoter methylation frequency in 308 CRC with *MLH1* protein expression (9.8%), which was lower than in 156 CRC without *MLH1* protein expression (69.8%, *P*<0.001). In CRC with loss of *MLH1* protein expression, the *MLH1* promoter methylation was significantly higher in sporadic CRC (69.8%) than in LS CRC (37.8%, *P = *0.026). In sporadic MSI-H CRC with loss of *MLH1* protein, the *MLH1* promoter methylation frequency was 86.3%. *MLH1* promoter methylation could explain more fraction of *MLH1* gene silencing in sporadic CRC than that in LS CRC. In this systematic review and meta-analysis, we can see that the highest frequency of *MLH1* promoter methylation was in MSI-H CRC with loss of *MLH1* protein, the following in sporadic CRC without *MLH1* protein expression, and the lowest in the sporadic CRC with *MLH1* protein expression.

The frequency of *MLH1* promoter methylation in *BRAF* mutated total CRC was 53.2%, significantly higher than in *BRAF* wild type total CRC 13.7% (*P = *0.001). In contrast, the *MLH1* promoter methylation frequency in *KRAS* mutated total CRC (14.0%) was significantly lower than in *KRAS* wild type total CRC (21.8%) (*P*<0.001). The largest population-based study [21] observed similar results, the frequencies of *MLH1* promoter methylation were 46.8% (51/109) in *BRAF* mutated CRC and 17.4% (97/559) *BRAF* wild type CRC (*P*<0.001); whereas, they were 15.5% (40/258) in *KRAS* mutated CRC and 26.2% (112/428) *KRAS* wild type CRC (*P = *0.001). In MSI CRC, similar results were also observed. The *MLH1* promoter methylation frequency in *BRAF* mutated MSI-H CRC (94.5%) was significantly higher than in *BRAF* wild MSI-H CRC (28.2%) (*P*<0.001). Whereas, the *MLH1* promoter methylation frequency in *KRAS* mutated MSI CRC (25.9%) was significantly lower than in *KRAS* wild MSI CRC (50.4%) (*P = *0.003). The following may explain the results: colon tumors progress by distinct genes of the RAS/RAF/MAP kinase pathway, depending on the genetic/epigenetic event underlying MMR deficiency (mutation and loss induced by *MLH1* promoter methylation). MSI-H tumors with MMR gene mutations (hereditary and sporadic forms) my preferentially target *KRAS*, whereas, MSI-H tumors with *MLH1* promoter methylation may preferentially target the *BRAF* gene [73]. In addition, the methylation of *MGMT* was associated with *KRAS* mutant CRC but not of *BRAF* mutant CRC could also support the results of our meta-analysis [20].

Our study suggested that the frequency of *MLH1* promoter methylation was higher in female, proximal tumor location, and poor differentiation. Study reported that MSI CRC had a very distinct clinicopathological phenotype, which was commonly mucinous, poorly differentiated, presenting at earlier Dukes’ stage, and in the proximal side of the colon [74]. MSI CRC was also commonly female and older at diagnosis. Moreover, in sporadic CRC, MSI was mainly caused by *MLH1* promoter methylation. Therefore, MSI CRC and *MLH1* promoter methylation CRC may have similar clinicopathological phenotype. However, the underlying mechanisms need to be investigated.

Heterogeneity persisted in our meta-analysis. The followings may explain the sources of heterogeneity. Firstly, *MLH1* promoter methylation was tested in different promoter regions. One study tested in “A” region; three studies tested in “C” region; other 15 studies did not supply specific regions tested. Additionally, various ages of the study subjects may also explain the heterogeneity. Genes of individual are progressively methylated with aging due to chromosomal instability [75]. However, only three of 19 studies provided the information of age of study subjects [16], [54], [55].

Although this meta-analysis provides some robust results, limitations also existed like all the meta-analysis. Firstly, dietary factors, smoking and drinking alcohol may affect the *MLH1* promoter methylation. Lack of these original data of the studies reviewed limited our further evaluation of their effect on *MLH1* promoter methylation [76], [77], [78]. Secondly, lacking of the original data limited our further evaluation of the interactions between the clinicopathological and molecular variables in CRC. Thirdly, the prevalence of *MLH1* promoter methylation in CRC may increase with aging. However, the majority of studies did not provide this information, which limited our further evaluate their effect on *MLH1* promoter methylation.

In summary, this systematic review and meta-analysis yield some conclusions: the *MLH1* methylation in total CRC was 20.3%; they were 18.7% in sporadic CRC and 16.4% in LS CRC, respectively; *MLH1* promoter methylation may be significantly associated with gender, tumor location, tumor differentiation, MSI, *MLH1* protein expression, and *BRAF* mutation.

## Supporting Information

Figure S1 **Forest figure for association of** ***MLHI*** **promoter methylation and MSI status in CRC tumors (MSI vs. MSS).**
(DOC)

Figure S2 **Funnel plot of the log (event rate) versus its standard error, for the frequency of** ***MLH1*** **promoter methylation in CRC tumors: (A) MSI and (B) MSI-H.**
(DOC)

Figure S3 **Funnel plot of the log odds ratio versus its standard error, for the frequency of** ***MLH1*** **promoter methylation in CRC tumors: Proximal vs. Distal.**
(DOC)

Table S1 **Pooled frequency of** ***MLH1*** **promoter methylation in colorectal cancer patients with other subgroup analysis.**
(DOC)

Text S1 **PRISMA Checklist.**
(DOC)

Text S2 **Review protocol.**
(DOC)
