# Supplementary material for: MLH1 Promoter Methylation Frequency in Colorectal Cancer Patients and Related Clinicopathological and Molecular Features
Source: PLoS One. 2013 Mar 29;8(3):e59064. doi: 10.1371/journal.pone.0059064 (PMC3612054; doi:10.1371/journal.pone.0059064)
Supplement: Table S1 — Pooled frequency of MLH1 promoter methylation in colorectal cancer patients with other subgroup analysis. (DOC) [file pone.0059064.s004.doc]

**Table S1 Pooled frequency of *MLH1* promoter methylation in colorectal cancer patients with other subgroup analysis**

| Classification | No. of | No. of | No. of | Pooled frequency | *P* | Heterogeneity | | Publication bias | |
| --- | --- | --- | --- | --- | --- | --- | --- | --- | --- |
|  | studies | detected cases | methylation cases | and 95%CI (%) |  | *I^2^* (%) | *P* | Begg’s test *P* | Egger’s test *P* |
| Ethnicity** |  |  |  |  | 0.296 |  |  |  |  |
| Europe& Australia | 9 | 2081 | 377 | 18.7(13.8-24.7)* | 0.000 | 87.564 | 0.000 | 1.000 | 0.795 |
| Asia | 2 | 75 | 17 | 23.0(14.8-34.0) | 0.001 | 24.902 | 0.249 | - | - |
| America | 6 | 1988 | 367 | 25.9(17.2-37.1)* | 0.000 | 92.732 | 0.000 | 0.348 | 0.272 |
| Mixed | 2 | 1440 | 244 | 13.4(6.7-25.2)* | 0.000 | 89.904 | 0.000 | - | - |
| Detecting methods** |  |  |  |  | 0.042 |  |  |  |  |
| MethyLight | 3 | 2352 | 356 | 13.0(8.8-18.8)* | 0.000 | 90.678 | 0.000 | 1.000 | 0.881 |
| MSP | 10 | 2637 | 502 | 20.3(16.0-25.4)* | 0.000 | 83.297 | 0.000 | 0.929 | 0.910 |
| Others | 6 | 595 | 147 | 25.7(16.0-25.4)* | 0.000 | 86.006 | 0.001 | 0.881 | 0.873 |
| Promoter region |  |  |  |  | 0.001 |  |  |  |  |
| A region tested | 1 | 151 | 101 | 66.9(59.0-73.9) | 0.000 | - | - | - | - |
| C region tested | 3 | 370 | 85 | 26.4(11.8-49.1)* | 0.042 | 91.255 | 0.000 | 0.602 | 0.967 |
| Materials ** |  |  |  |  | 0.751 |  |  |  |  |
| Fresh-frozen specimens | 6 | 1669 | 285 | 18.4(12.5-26.3)* | 0.000 | 88.744 | 0.000 | 0.117 | 0.543 |
| Formalin-fixed, paraffin-embedded tissues | 3 | 1151 | 154 | 16.5(9.4-27.4)* | 0.000 | 87.502 | 0.000 | 0.573 | 0.996 |

* Random effect estimate.

** It is only pooled data with total colorectal cancer.
