# Supplementary material for: MLH1 Promoter Methylation Frequency in Colorectal Cancer Patients and Related Clinicopathological and Molecular Features
Source: PLoS One. 2013 Mar 29;8(3):e59064. doi: 10.1371/journal.pone.0059064 (PMC3612054; doi:10.1371/journal.pone.0059064)
Supplement: Text S2 — Review protocol. (DOC) [file pone.0059064.s006.doc]

| **Title**  **Text S2** Study protocol for *MLH1* Promoter Methylation Frequency in Colorectal Cancer Patients and Related Clinicopathological and Molecular Features. | | |
| --- | --- | --- |
| Title | | *MLH1* Promoter Methylation Frequency in Colorectal Cancer Patients and Related Clinicopathological and Molecular Features |
| **Protocol information:** | | |
| Authors* | | Xia Li, Xiaoping Yao, Yibaina Wang, Fulan Hu, FanWang, Liying Jiang, Yupeng Liu, Da Wang, Guizhi Sun, Yashuang Zhao  **Principal Investigator:** Zhao Yashuang: M.D., Ph.D. ^1^Department of Epidemiology, School of Public Health, Harbin Medical University, Harbin, Heilongjiang Province, P. R. China. ^2^Department of Evidence-based medicine, School of Public Health, Harbin Medical University, Harbin, Heilongjiang Province, P. R. China. |
| Sources of support | | This work was supported by grants from National Natural Science Foundation of China for Priority Areas (NSFC 30972538), Education Bureau of Heilongjiang Province (11531100), and the Graduate Foundation, supported by the Scientific Research of Heilongjiang Province (YJSCX2009-224HLJ) and Harbin Medical University (HCXB2009009). |
| What’s new | | As we know, multiple independent studies have reported *MLH1* promoter methylation in sporadic colorectal cancers and Lynch syndrome and their association with different clinicalpathological and molecular characteristics. However, the results are inconsistent. In this study, we conducted a systematic review and meta-analysis to accurately estimate the frequency of *MLH1* promoter methylation in sporadic colorectal cancers and Lynch syndrome, and the associations between *MLH1* promoter methylation and clinicopathological/molecular characteristics of CRC. |
| **The protocol:** | | |
| Background* | | The associations between *MLH1* promoter methylation and other clinicopathological and molecular characteristics of CRC such as tumor location, tumor staging, tumor differentiation, family history, MSI, and *MLH1* protein expression were also widely studied. However, the results are inconsistent. |
| Objectives* | | To describe the frequency of *MLH1* promoter methylation in colorectal cancer (CRC); to explore the associations between *MLH1* promoter methylation and clinicopathological and molecular factors using a systematic review and meta-analysis. |
| **Methods** | | |
| **Criteria for selecting studies for this review:**  The inclusion/exclusion criteria were as follows: (1) papers on *MLH1* promoter methylation in unselected CRC were included. In contrast, papers that selected subgroups were excluded (such as selected based on age, tumor staging and ulcerative colitis-associated CRC); (2) sporadic CRC and/or LS related CRC remained as specific selected groups, often stratified by MSI status and/or *MLH1* expression loss; (3) data regarding the DNA methylation of tumor tissue of CRC were included in the pooled analysis, whereas data regarding the DNA methylation of normal colonic mucosa, serum, and peripheral blood leukocyte of CRC were excluded; (4) studies that investigated multiple CRCs were excluded; (5) case reports were excluded; (6) repetitive reports were unified by using the latest or the largest edition; (7) paper with insufficient or duplicated data were excluded. | | |
| Types of studies* | | Descriptive research |
| Types of participants* | | Colorectal cancer patients |
| Types of interventions* | | n/a |
| Types of outcome measures* | | The pooled frequency of *MLH1* promoter methylation and 95% confidence intervals (95% CI) were estimated. The pooled OR was estimated for the association between *MLH1* promoter methylation and clinicopathological, molecular features. |
| Search methods for identification of studies*: | | |
| We conducted a systematic literature search using PubMed and Embase from January 1, 1997 to September 7, 2012 to identify all the relevant English-language articles. The following keywords were used: “methylation” and “*MLH1*” and “promoter” and “colorectal cancer” and/or “carcinoma” or “tumor” or “neoplasm”. We also hand-searched the reference lists of the retrieved articles and reviews for additional articles. | | |
| Data collection and analysis* | | |
| **Data extraction**  Two authors (X. and X.P) independently conducted literature searches to identify all possible papers that met the inclusion criteria. Disagreements were settled by consensus or a third review (Y.B.N) for adjudication. The following information were extracted from every eligible study: authors, publication year, continent, country, patient source, sample size, methylation detecting method, positive frequency, gender, family history, tumor location (proximal and distal), tumor staging, and promoter regions. | | |
| **Meta-analysis**  The pooled frequency of *MLH1* promoter methylation and 95% confidence intervals (95% CI) were estimated. The frequency of *MLH1* promoter methylation was compared in different tumor characteristics. Heterogeneity among studies was evaluated with Cochran’s Q test[1] and the *I^2^* statistic [2,3]. When heterogeneity was not an issue (*I^2^* values < 50%), a fixed effect model was used to calculate parameters. If there was substantial heterogeneity (*I^2^* values ≥ 50%), a random-effects model was used to pool data and attempt to identify potential sources of heterogeneity based on subgroup analyses. The pooled OR was estimated for the association between *MLH1* promoter methylation and clinicopathological, molecular features. *P* values tailed less than 0.05 were considered statistically significant.  Publication bias was evaluated with funnel plot, Begg’s rank correlation [4], and Egger’s regression [5]. If publication bias existed, the trim and fill method was used to adjust the pooled frequency, pooled OR and 95% CI [6]. Data were calculated with Comprehensive Meta-Analysis V2. | | |
| Acknowledgements: | | |
| References: | | |
| **Included studies**  752 relevant articles were identified for initial review according to the inclusion and exclusion criteria. After screening, information for 10528 individuals from 96 studies was reviewed and included in the meta-analyses.  1. Bouzourene H, Taminelli L, Chaubert P, Monnerat C, Seelentag W, et al. (2006) A cost-effective algorithm for hereditary nonpolyposis colorectal cancer detection. Am J Clin Pathol 125: 823-831.  2. Lind GE, Thorstensen L, Lovig T, Meling GI, Hamelin R, et al. (2004) A CpG island hypermethylation profile of primary colorectal carcinomas and colon cancer cell lines. Mol Cancer 3: 28.  3. Lee S, Hwang KS, Lee HJ, Kim JS, Kang GH (2004) Aberrant CpG island hypermethylation of multiple genes in colorectal neoplasia. Lab Invest 84: 884-893.  4. Domingo E, Espin E, Armengol M, Oliveira C, Pinto M, et al. (2004) Activated BRAF targets proximal colon tumors with mismatch repair deficiency and MLH1 inactivation. Genes Chromosomes Cancer 39: 138-142.  5. Iacopetta B, Grieu F, Li W, Ruszkiewicz A, Caruso M, et al. (2006) APC gene methylation is inversely correlated with features of the CpG island methylator phenotype in colorectal cancer. Int J Cancer 119: 2272-2278.  6. Raedle J, Trojan J, Brieger A, Weber N, Schafer D, et al. (2001) Bethesda guidelines: relation to microsatellite instability and MLH1 promoter methylation in patients with colorectal cancer. Ann Intern Med 135: 566-576.  7. Deng G, Bell I, Crawley S, Gum J, Terdiman JP, et al. (2004) BRAF mutation is frequently present in sporadic colorectal cancer with methylated hMLH1, but not in hereditary nonpolyposis colorectal cancer. Clin Cancer Res 10: 191-195.  8. Lubomierski N, Plotz G, Wormek M, Engels K, Kriener S, et al. (2005) BRAF mutations in colorectal carcinoma suggest two entities of microsatellite-unstable tumors. Cancer 104: 952-961.  9. Potocnik U, Glavac D, Golouh R, Ravnik-Glavac M (2001) Causes of microsatellite instability in colorectal tumors: implications for hereditary non-polyposis colorectal cancer screening. Cancer Genet Cytogenet 126: 85-96.  10. Kim JC, Lee KH, Ka IH, Koo KH, Roh SA, et al. (2004) Characterization of mutator phenotype in familial colorectal cancer patients not fulfilling amsterdam criteria. Clin Cancer Res 10: 6159-6168.  11. Yan HL, Hao LQ, Jin HY, Xing QH, Xue G, et al. (2008) Clinical features and mismatch repair genes analyses of Chinese suspected hereditary non-polyposis colorectal cancer: a cost-effective screening strategy proposal. Cancer Sci 99: 770-780.  12. Cai G, Xu Y, Lu H, Shi Y, Lian P, et al. (2008) Clinicopathologic and molecular features of sporadic microsatellite- and chromosomal-stable colorectal cancers. Int J Colorectal Dis 23: 365-373.  13. Park IJ, Kim HC, Yoon YS, Yu CS, Jang SJ, et al. (2007) Clinicopathological characteristics of colorectal cancer with family history: an evaluation of family history as a predictive factor for microsatellite instability. J Korean Med Sci 22 Suppl: S91-97.  14. Rahner N, Friedrichs N, Steinke V, Aretz S, Friedl W, et al. (2008) Coexisting somatic promoter hypermethylation and pathogenic MLH1 germline mutation in Lynch syndrome. J Pathol 214: 10-16.  15. Anacleto C, Leopoldino AM, Rossi B, Soares FA, Lopes A, et al. (2005) Colorectal cancer "methylator phenotype": fact or artifact? Neoplasia 7: 331-335.  16. Toyota M, Ahuja N, Ohe-Toyota M, Herman JG, Baylin SB, et al. (1999) CpG island methylator phenotype in colorectal cancer. Proc Natl Acad Sci U S A 96: 8681-8686.  17. Hawkins N, Norrie M, Cheong K, Mokany E, Ku SL, et al. (2002) CpG island methylation in sporadic colorectal cancers and its relationship to microsatellite instability. Gastroenterology 122: 1376-1387.  18. Lee S, Cho NY, Yoo EJ, Kim JH, Kang GH (2008) CpG island methylator phenotype in colorectal cancers: comparison of the new and classic CpG island methylator phenotype marker panels. Arch Pathol Lab Med 132: 1657-1665.  19. Koopman M, Kortman GA, Mekenkamp L, Ligtenberg MJ, Hoogerbrugge N, et al. (2009) Deficient mismatch repair system in patients with sporadic advanced colorectal cancer. Br J Cancer 100: 266-273.  20. Grady WM, Rajput A, Lutterbaugh JD, Markowitz SD (2001) Detection of aberrantly methylated hMLH1 promoter DNA in the serum of patients with microsatellite unstable colon cancer. Cancer Res 61: 900-902.  21. Jin HY, Liu X, Li VK, Ding Y, Yang B, et al. (2008) Detection of mismatch repair gene germline mutation carrier among Chinese population with colorectal cancer. BMC Cancer 8: 44.  22. Yamamoto H, Min Y, Itoh F, Imsumran A, Horiuchi S, et al. (2002) Differential involvement of the hypermethylator phenotype in hereditary and sporadic colorectal cancers with high-frequency microsatellite instability. Genes Chromosomes Cancer 33: 322-325.  23. Chan AO, Soliman AS, Zhang Q, Rashid A, Bedeir A, et al. (2005) Differing DNA methylation patterns and gene mutation frequencies in colorectal carcinomas from Middle Eastern countries. Clin Cancer Res 11: 8281-8287.  24. Kumar K, Brim H, Giardiello F, Smoot DT, Nouraie M, et al. (2009) Distinct BRAF (V600E) and KRAS mutations in high microsatellite instability sporadic colorectal cancer in African Americans. Clin Cancer Res 15: 1155-1161.  25. Oliveira C, Westra JL, Arango D, Ollikainen M, Domingo E, et al. (2004) Distinct patterns of KRAS mutations in colorectal carcinomas according to germline mismatch repair defects and hMLH1 methylation status. Hum Mol Genet 13: 2303-2311.  26. Bettstetter M, Dechant S, Ruemmele P, Grabowski M, Keller G, et al. (2007) Distinction of hereditary nonpolyposis colorectal cancer and sporadic microsatellite-unstable colorectal cancer through quantification of MLH1 methylation by real-time PCR. Clin Cancer Res 13: 3221-3228.  27. Maekawa M, Sugano K, Kashiwabara H, Ushiama M, Fujita S, et al. (1999) DNA methylation analysis using bisulfite treatment and PCR-single-strand conformation polymorphism in colorectal cancer showing microsatellite instability. Biochem Biophys Res Commun 262: 671-676.  28. Kuismanen SA, Holmberg MT, Salovaara R, Schweizer P, Aaltonen LA, et al. (1999) Epigenetic phenotypes distinguish microsatellite-stable and -unstable colorectal cancers. Proc Natl Acad Sci U S A 96: 12661-12666.  29. Joensuu EI, Abdel-Rahman WM, Ollikainen M, Ruosaari S, Knuutila S, et al. (2008) Epigenetic signatures of familial cancer are characteristic of tumor type and family category. Cancer Res 68: 4597-4605.  30. Suehiro Y, Wong CW, Chirieac LR, Kondo Y, Shen L, et al. (2008) Epigenetic-genetic interactions in the APC/WNT, RAS/RAF, and P53 pathways in colorectal carcinoma. Clin Cancer Res 14: 2560-2569.  31. Samowitz WS, Albertsen H, Herrick J, Levin TR, Sweeney C, et al. (2005) Evaluation of a large, population-based sample supports a CpG island methylator phenotype in colon cancer. Gastroenterology 129: 837-845.  32. Arnold CN, Goel A, Compton C, Marcus V, Niedzwiecki D, et al. (2004) Evaluation of microsatellite instability, hMLH1 expression and hMLH1 promoter hypermethylation in defining the MSI phenotype of colorectal cancer. Cancer Biol Ther 3: 73-78.  33. Boardman LA, Lanier AP, French AJ, Schowalter KV, Burgart LJ, et al. (2007) Frequency of defective DNA mismatch repair in colorectal cancer among the Alaska Native people. Cancer Epidemiol Biomarkers Prev 16: 2344-2350.  34. Ogawa T, Yoshida T, Tsuruta T, Saigenji K, Okayasu I (2006) Genetic instability on chromosome 17 in the epithelium of non-polypoid colorectal carcinomas compared to polypoid lesions. Cancer Sci 97: 1335-1342.  35. van Puijenbroek M, Middeldorp A, Tops CM, van Eijk R, van der Klift HM, et al. (2008) Genome-wide copy neutral LOH is infrequent in familial and sporadic microsatellite unstable carcinomas. Fam Cancer 7: 319-330.  36. Strazzullo M, Cossu A, Baldinu P, Colombino M, Satta MP, et al. (2003) High-resolution methylation analysis of the hMLH1 promoter in sporadic endometrial and colorectal carcinomas. Cancer 98: 1540-1546.  37. Ricciardiello L, Ceccarelli C, Angiolini G, Pariali M, Chieco P, et al. (2005) High thymidylate synthase expression in colorectal cancer with microsatellite instability: implications for chemotherapeutic strategies. Clin Cancer Res 11: 4234-4240.  38. Yearsley M, Hampel H, Lehman A, Nakagawa H, de la Chapelle A, et al. (2006) Histologic features distinguish microsatellite-high from microsatellite-low and microsatellite-stable colorectal carcinomas, but do not differentiate germline mutations from methylation of the MLH1 promoter. Hum Pathol 37: 831-838.  39. Kamory E, Kolacsek O, Otto S, Csuka O (2003) hMLH1 and hMSH2 somatic inactivation mechanisms in sporadic colorectal cancer patients. Pathol Oncol Res 9: 236-241.  40. Julie C, Tresallet C, Brouquet A, Vallot C, Zimmermann U, et al. (2008) Identification in daily practice of patients with Lynch syndrome (hereditary nonpolyposis colorectal cancer): revised Bethesda guidelines-based approach versus molecular screening. Am J Gastroenterol 103: 2825-2835; quiz 2836.  41. Mori Y, Yin J, Sato F, Sterian A, Simms LA, et al. (2004) Identification of genes uniquely involved in frequent microsatellite instability colon carcinogenesis by expression profiling combined with epigenetic scanning. Cancer Res 64: 2434-2438.  42. Sanchez JA, Vogel JD, Kalady MF, Bronner MP, Skacel M, et al. (2008) Identifying Lynch syndrome: we are all responsible. Dis Colon Rectum 51: 1750-1756.  43. Truninger K, Menigatti M, Luz J, Russell A, Haider R, et al. (2005) Immunohistochemical analysis reveals high frequency of PMS2 defects in colorectal cancer. Gastroenterology 128: 1160-1171.  44. Mas S, Lafuente MJ, Crescenti A, Trias M, Ballesta A, et al. (2007) Lower specific micronutrient intake in colorectal cancer patients with tumors presenting promoter hypermethylation in p16(INK4a), p4(ARF) and hMLH1. Anticancer Res 27: 1151-1156.  45. Yiu R, Qiu H, Lee SH, Garcia-Aguilar J (2005) Mechanisms of microsatellite instability in colorectal cancer patients in different age groups. Dis Colon Rectum 48: 2061-2069.  46. Shi X, Li J, Zhao C, Lv S, Xu G (2006) Methylation analysis of hMLH1 gene promoter by a bisulfite-sensitive single-strand conformation polymorphism-capillary electrophoresis method. Biomed Chromatogr 20: 815-820.  47. Shannon BA, Iacopetta BJ (2001) Methylation of the hMLH1, p16, and MDR1 genes in colorectal carcinoma: associations with clinicopathological features. Cancer Lett 167: 91-97.  48. Menigatti M, Di Gregorio C, Borghi F, Sala E, Scarselli A, et al. (2001) Methylation pattern of different regions of the MLH1 promoter and silencing of gene expression in hereditary and sporadic colorectal cancer. Genes Chromosomes Cancer 31: 357-361.  49. Mokarram P, Naghibalhossaini F, Saberi Firoozi M, Hosseini SV, Izadpanah A, et al. (2008) Methylenetetrahydrofolate reductase C677T genotype affects promoter methylation of tumor-specific genes in sporadic colorectal cancer through an interaction with folate/vitamin B12 status. World J Gastroenterol 14: 3662-3671.  50. de Vogel S, Weijenberg MP, Herman JG, Wouters KA, de Goeij AF, et al. (2009) MGMT and MLH1 promoter methylation versus APC, KRAS and BRAF gene mutations in colorectal cancer: indications for distinct pathways and sequence of events. Ann Oncol 20: 1216-1222.  51. Salahshor S, Koelble K, Rubio C, Lindblom A (2001) Microsatellite Instability and hMLH1 and hMSH2 expression analysis in familial and sporadic colorectal cancer. Lab Invest 81: 535-541.  52. Ghimenti C, Tannergard P, Wahlberg S, Liu T, Giulianotti PG, et al. (1999) Microsatellite instability and mismatch repair gene inactivation in sporadic pancreatic and colon tumours. Br J Cancer 80: 11-16.  53. Noda H, Kato Y, Yoshikawa H, Arai M, Togashi K, et al. (2005) Microsatellite instability caused by hMLH1 promoter methylation increases with tumor progression in right-sided sporadic colorectal cancer. Oncology 69: 354-362.  54. Vilkin A, Niv Y, Nagasaka T, Morgenstern S, Levi Z, et al. (2009) Microsatellite instability, MLH1 promoter methylation, and BRAF mutation analysis in sporadic colorectal cancers of different ethnic groups in Israel. Cancer 115: 760-769.  55. Poynter JN, Siegmund KD, Weisenberger DJ, Long TI, Thibodeau SN, et al. (2008) Molecular characterization of MSI-H colorectal cancer by MLHI promoter methylation, immunohistochemistry, and mismatch repair germline mutation screening. Cancer Epidemiol Biomarkers Prev 17: 3208-3215.  56. Whitehall VL, Wynter CV, Walsh MD, Simms LA, Purdie D, et al. (2002) Morphological and molecular heterogeneity within nonmicrosatellite instability-high colorectal cancer. Cancer Res 62: 6011-6014.  57. Fox EJ, Leahy DT, Geraghty R, Mulcahy HE, Fennelly D, et al. (2006) Mutually exclusive promoter hypermethylation patterns of hMLH1 and O6-methylguanine DNA methyltransferase in colorectal cancer. J Mol Diagn 8: 68-75.  58. Maestro ML, Vidaurreta M, Sanz-Casla MT, Rafael S, Veganzones S, et al. (2007) Role of the BRAF mutations in the microsatellite instability genetic pathway in sporadic colorectal cancer. Ann Surg Oncol 14: 1229-1236.  59. Hampel H, Frankel WL, Martin E, Arnold M, Khanduja K, et al. (2005) Screening for the Lynch syndrome (hereditary nonpolyposis colorectal cancer). N Engl J Med 352: 1851-1860.  60. Jensen LH, Lindebjerg J, Byriel L, Kolvraa S, Cruger DG (2008) Strategy in clinical practice for classification of unselected colorectal tumours based on mismatch repair deficiency. Colorectal Dis 10: 490-497.  61. Karpinski P, Ramsey D, Grzebieniak Z, Sasiadek MM, Blin N (2008) The CpG island methylator phenotype correlates with long-range epigenetic silencing in colorectal cancer. Mol Cancer Res 6: 585-591.  62. Cunningham JM, Kim CY, Christensen ER, Tester DJ, Parc Y, et al. (2001) The frequency of hereditary defective mismatch repair in a prospective series of unselected colorectal carcinomas. Am J Hum Genet 69: 780-790.  63. Belshaw NJ, Elliott GO, Williams EA, Bradburn DM, Mills SJ, et al. (2004) Use of DNA from human stools to detect aberrant CpG island methylation of genes implicated in colorectal cancer. Cancer Epidemiol Biomarkers Prev 13: 1495-1501.  64. Paya A, Alenda C, Perez-Carbonell L, Rojas E, Soto JL, et al. (2009) Utility of p16 immunohistochemistry for the identification of Lynch syndrome. Clin Cancer Res 15: 3156-3162.  65. Prabhu JS, Korlimarla A, Banerjee A, Wani S, K P, et al. (2009) Gene-specific methylation: potential markers for colorectal cancer. Int J Biol Markers 24: 57-62.  66. Woods MO, Hyde AJ, Curtis FK, Stuckless S, Green JS, et al. (2005) High frequency of hereditary colorectal cancer in Newfoundland likely involves novel susceptibility genes. Clin Cancer Res 11: 6853-6861.  67. Asaka S, Arai Y, Nishimura Y, Yamaguchi K, Ishikubo T, et al. (2009) Microsatellite instability-low colorectal cancer acquires a KRAS mutation during the progression from Dukes' A to Dukes' B. Carcinogenesis 30: 494-499.  68. Ogino S, Kawasaki T, Kirkner GJ, Kraft P, Loda M, et al. (2007) Evaluation of markers for CpG island methylator phenotype (CIMP) in colorectal cancer by a large population-based sample. J Mol Diagn 9: 305-314.  69. Brandes JC, van Engeland M, Wouters KA, Weijenberg MP, Herman JG (2005) CHFR promoter hypermethylation in colon cancer correlates with the microsatellite instability phenotype. Carcinogenesis 26: 1152-1156.  70. Nakagawa H, Nagasaka T, Cullings HM, Notohara K, Hoshijima N, et al. (2009) Efficient molecular screening of Lynch syndrome by specific 3' promoter methylation of the MLH1 or BRAF mutation in colorectal cancer with high-frequency microsatellite instability. Oncol Rep 21: 1577-1583.  71. South CD, Yearsley M, Martin E, Arnold M, Frankel W, et al. (2009) Immunohistochemistry staining for the mismatch repair proteins in the clinical care of patients with colorectal cancer. Genet Med 11: 812-817.  72. Baek YH, Chang E, Kim YJ, Kim BK, Sohn JH, et al. (2009) Stool methylation-specific polymerase chain reaction assay for the detection of colorectal neoplasia in Korean patients. Dis Colon Rectum 52: 1452-1459; discussion 1459-1463.  73. M. Mirchev IK, P. Kahl, R. Büttner, L. Angelova, B. Manevska, T., and Kadyiska. (2007) Epigenetic silencing of MLH1 and p16INK and their relation to certain clinicopathological features in patients with colorectal cancer. Journal of IMAB - Annual Proceeding (Scientific Papers): 95-96.  74. Barault L, Charon-Barra C, Jooste V, de la Vega MF, Martin L, et al. (2008) Hypermethylator phenotype in sporadic colon cancer: study on a population-based series of 582 cases. Cancer Res 68: 8541-8546.  75. Tunca B, Pedroni M, Cecener G, Egeli U, Borsi E, et al. (2010) Analysis of mismatch repair gene mutations in Turkish HNPCC patients. Fam Cancer 9: 365-376.  76. Kim JC, Choi JS, Roh SA, Cho DH, Kim TW, et al. (2010) Promoter methylation of specific genes is associated with the phenotype and progression of colorectal adenocarcinomas. Ann Surg Oncol 17: 1767-1776.  77. Chang SC, Lin PC, Yang SH, Wang HS, Liang WY, et al. (2011) Taiwan hospital-based detection of Lynch syndrome distinguishes 2 types of microsatellite instabilities in colorectal cancers. Surgery 147: 720-728.  78. Perez-Carbonell L, Alenda C, Paya A, Castillejo A, Barbera VM, et al. (2010) Methylation analysis of MLH1 improves the selection of patients for genetic testing in Lynch syndrome. J Mol Diagn 12: 498-504.  79. Bouzourene H, Hutter P, Losi L, Martin P, Benhattar J (2010) Selection of patients with germline MLH1 mutated Lynch syndrome by determination of MLH1 methylation and BRAF mutation. Fam Cancer 9: 167-172.  80. Woods MO, Younghusband HB, Parfrey PS, Gallinger S, McLaughlin J, et al. (2010) The genetic basis of colorectal cancer in a population-based incident cohort with a high rate of familial disease. Gut 59: 1369-1377.  81. Rasuck CG, Leite SM, Komatsuzaki F, Ferreira AC, Oliveira VC, et al. (2011) Association between methylation in mismatch repair genes, V600E BRAF mutation and microsatellite instability in colorectal cancer patients. Mol Biol Rep 39: 2553-2560.  82. Karpinski P, Myszka A, Ramsey D, Kielan W, Sasiadek MM (2011) Detection of viral DNA sequences in sporadic colorectal cancers in relation to CpG island methylation and methylator phenotype. Tumour Biol 32: 653-659.  83. Aoyagi H, Iida S, Uetake H, Ishikawa T, Takagi Y, et al. (2011) Effect of classification based on combination of mutation and methylation in colorectal cancer prognosis. Oncol Rep 25: 789-794.  84. Zlobec I, Bihl MP, Foerster A, Rufle A, Terracciano L, et al. (2012) Stratification and Prognostic Relevance of Jass's Molecular Classification of Colorectal Cancer. Front Oncol 2: 7.  85. Network TCGA (2012) Comprehensive molecular characterization of human colon and rectal cancer. Nature 487: 330-337.  86. Urso E, Agostini M, Pucciarelli S, Rugge M, Bertorelle R, et al. (2012) Clinical and molecular detection of inherited colorectal cancers in northeast Italy: a first prospective study of incidence of Lynch syndrome and MUTYH-related colorectal cancer in Italy. Tumour Biol 33: 857-864.  87. Lee KH, Lee JS, Nam JH, Choi C, Lee MC, et al. (2011) Promoter methylation status of hMLH1, hMSH2, and MGMT genes in colorectal cancer associated with adenoma-carcinoma sequence. Langenbecks Arch Surg 396: 1017-1026.  88. Lin CH, Lin JK, Chang SC, Chang YH, Chang HM, et al. (2011) Molecular profile and copy number analysis of sporadic colorectal cancer in Taiwan. J Biomed Sci 18: 36.  89. Sanchez JA, Dejulius KL, Bronner M, Church JM, Kalady MF (2011) Relative role of methylator and tumor suppressor pathways in ulcerative colitis-associated colon cancer. Inflamm Bowel Dis 17: 1966-1970.  90. Miladi-Abdennadher I, Abdelmaksoud-Damak R, Ayadi L, Khabir A, Frikha F, et al. (2011) Aberrant methylation of hMLH1 and p16INK4a in Tunisian patients with sporadic colorectal adenocarcinoma. Biosci Rep 31: 257-264.  91. Huang Q, Huang JF, Zhang B, Baum L, Fu WL (2012) Methylation variable position profiles of hMLH1 promoter CpG islands in human sporadic colorectal carcinoma. Diagn Mol Pathol 21: 24-33.  92. Gausachs M, Mur P, Corral J, Pineda M, Gonzalez S, et al. (2012) MLH1 promoter hypermethylation in the analytical algorithm of Lynch syndrome: a cost-effectiveness study. Eur J Hum Genet 20: 762-768.  93. Gay LJ, Arends MJ, Mitrou PN, Bowman R, Ibrahim AE, et al. (2011) MLH1 promoter methylation, diet, and lifestyle factors in mismatch repair deficient colorectal cancer patients from EPIC-Norfolk. Nutr Cancer 63: 1000-1010.  94. Canard G, Lefevre JH, Colas C, Coulet F, Svrcek M, et al. (2012) Screening for Lynch syndrome in colorectal cancer: are we doing enough? Ann Surg Oncol 19: 809-816.  95. Vasovcak P, Pavlikova K, Sedlacek Z, Skapa P, Kouda M, et al. (2011) Molecular genetic analysis of 103 sporadic colorectal tumours in Czech patients. PLoS One 6: e24114.  96. Alemayehu A, Sebova K, Fridrichova I (2008) Redundant DNA methylation in colorectal cancers of Lynch-syndrome patients. Genes Chromosomes Cancer 47: 906-914. | | |
| Other references: | 1. DerSimonian R, Laird N (1986) Meta-analysis in clinical trials. Control Clin Trials 7: 177-188.  2. Higgins JP, Thompson SG, Deeks JJ, Altman DG (2003) Measuring inconsistency in meta-analyses. BMJ 327: 557-560.  3. DerSimonian R (1996) Meta-analysis in the design and monitoring of clinical trials. Stat Med 15: 1237-1248; discussion 1249-1252.  4. Begg CB, Mazumdar M (1994) Operating characteristics of a rank correlation test for publication bias. Biometrics 50: 1088-1101.  5. Egger M, Davey Smith G, Schneider M, Minder C (1997) Bias in meta-analysis detected by a simple, graphical test. BMJ 315: 629-634.  6. Duval S, Tweedie R (2000) Trim and fill: A simple funnel-plot-based method of testing and adjusting for publication bias in meta-analysis. Biometrics 56: 455-463. | |
| Tables and figures: | | |
| Tables | Table 1. Pooled frequency of *MLH1* promoter methylation in colorectal cancer patients with different clinicopathological features.  Table 2. Pooled frequency of *MLH1* promoter methylation in colorectal cancer patients with different molecular features.  Table 3. Pooled associations between *MLH1* promoter methylation and clinicopathological and molecular features. | |
| Figures | Figure1. Flow diagram of study selection.  Figure2. The pooled frequency of *MLH1* promoter methylation in CRC. | |
| Supporting Information | | |
| Additional Figures | Figure S1. Forest figure for association of *MLHI* promoter methylation and MSI status in CRC tumors (MSI vs. MSS).  Figure S2. Funnel plot of the log (event rate) versus its standard error, for the frequency of *MLH1* promoter methylation in CRC tumors: (A) MSI and (B) MSI-H.  Figure S3. Funnel plot of the log odds ratio versus its standard error, for the frequency of *MLH1* promoter methylation in CRC tumors: Proximal vs. Distal. | |
| Additional tables | Table S1. Pooled frequency of *MLH1* promoter methylation in colorectal cancer patients with other subgroup analysis. | |
| Checklist | Text S1. PRISMA Checklist. | |
| Protocol | Text S2. Protocol for the systematic review. | |
| About the article: | | |
| Contributions of authors | | Conceived and designed the experiments: Yashuang Zhao;  Performed the experiments: Xia Li, Xiaoping Yao, Yibaina Wang;  Analyzed the data: Fan Wang, Liying Jiang, Yupeng Liu;  Contributed reagents/materials/analysis tools: Guizhi Sun, Da Wang;  Wrote the paper: Xia Li, Fulan Hu, Yashuang Zhao. |
| Declarations of interest* | | We promise that there is no conflict of interest (such as employment, consultancies, stock ownership, honoraria, etc.) for this paper. |
| Published notes | | n/a |
